# Solution Equilibria between Aluminum(III) Ion and L-histidine or L-tyrosine

**DOI:** 10.1155/MBD.2002.235

**Published:** 2002

**Authors:** Predrag Djurdjevic, Ratomir Jelic, Dragana Dzajevic, Mirjana Cvijovic

**Affiliations:** 1 Faculty of Science Institute of Chemistry P.O. Box 60 Kragujevac 34000 Serbia and Montenegro; 2 Copper and Aluminium Smelting Factory Sevojno Uzice 31000 Serbia and Montenegro

## Abstract

Toxic effects due to high aluminum body loads were observed in a number of conditions following ingestion of Al-containing antacids. Bio-availability of aluminum depends not only on the solubility of the ingested salt but also on the physico-chemical properties of the soluble Al complexes formed in body fluids. Amino acids may, upon interaction with Al-salts, form absorbable Al-complexes. Hence, complex formation equilibria between Al^3+^ and either, L- histidine or L-tyrosine were studied by glass electrode potentiometric (0.1 mol/L LiCl ionic medium, 298 K), proton NMR and uv spectrophotometric measurements. Non linear least squares treatment of the potentiometric data indicates that in the concentration ranges: 0.5≤C_A1_≤2.0 ; 1.0≤C_His_≤10.0; 2.5≤PH≤6.5, in Al^3+^ + His solutions, the following complexes (with log overall stability constants given in parenthesis) are formed: Al(HHis)^3+^(12.21±0.08); Al(His)^2+^, (7.25±0.08); and Al(HHis)His^2+^, (20.3±0.1). In Al^3+^ + Tyr solutions in the concentration range 1.0≤C_Tyr_≤3.0 mmol/L and ligand to metal concentration ratio from 2:1 to 3:1, in the pH interval from 3.0 to 6.5 the formation of the following complexes was detected: Al(HTyr)^2+^, (12.72±0.09); Al(Tyr)^2+^, (10.16±0.03) and Al(OH)_2_Tyr , (2.70±0.05). Proton NMR data indicate that in Al(His)^2+^ complex histidine acts as a monodentate ligand but its bidentate coordination is possible with carboxylate oxygen and imidazole 1-nitrogen as donors. In Al(HTyr)^3+^ complex tyrosine is a monodentate ligand with carboxylate oxygen as donor. The mechanism of the formation of complexes in solution is discussed as well as their possible role in aluminum toxicity.


### SOLUTION EQUILIBRIA BETWEEN ALUMINUM(III) ION AND L-HISTIDINE OR L-TYROSINE

Predrag Djurdjevic*[1], Ratomir Jelic[1], Dragana Dzajevic[1] and Mirjana Cvijovic[2]

[1] Faculty of Science, Institute of Chemistry, P.O. Box 60, 34000 Kragujevac, Yugoslavia <preza@ptt.yu>
[2] Copper and Aluminium Smelting Factory, Sevojno, 31000 Uzice, Yugoslavia

**Abstract**

Toxic effects due to high aluminum body loads were observed in a number of conditions following ingestion of Al-containing antacids. Bio-availability of aluminum depends not only on the solubility of the ingested salt but also on the physico-chemical properties of the soluble Al complexes formed in body fluids. Amino acids may, upon interaction with Al-salts, form absorbable Al-complexes. Hence, complex formation equilibria between $Al^{3+}$ and either, L- histidine or L-tyrosine were studied by glass electrode potentiometric (0.1 mol/L LiCl ionic medium, 298 K), proton NMR and uv spectrophotometric measurements. Non linear least squares treatment of the potentiometric data indicates that in the concentration ranges: $0.5 \le C_{Al} \le 2.0$; $1.0 \le C_{His} \le 10.0$; $2.5 \le pH \le 6.5$, in $Al^{3+}$ + His solutions, the following complexes (with log overall stability constants given in parenthesis) are formed: $Al(HHis)^{3+}$ (12.21 ± 0.08); $Al(His)^{2+}$, (7.25 ± 0.08); and $Al(HHis)His^{2+}$, (20.3 ± 0.1). In $Al^{3+}$ + Tyr solutions in the concentration range $1.0 \le C_{Tyr} \le 3.0$ mmol/L and ligand to metal concentration ratio from 2:1 to 3:1, in the pH interval from 3.0 to 6.5 the formation of the following complexes was detected: $Al(HTyr)^{2+}$, (12.72 ± 0.09); $Al(Tyr)^{2+}$, (10.16 ± 0.03) and $Al(OH)_2Tyr$, (2.70 ± 0.05). Proton NMR data indicate that in $Al(His)^{2+}$ complex histidine acts as a monodentate ligand but its bidentate coordination is possible with carboxylate oxygen and imidazole 1-nitrogen as donors. In $Al(HTyr)^{3+}$ complex tyrosine is a monodentate ligand with carboxylate oxygen as donor. The mechanism of the formation of complexes in solution is discussed as well as their possible role in aluminum toxicity.

### Introduction

Aluminum is generally regarded as toxic or detrimental element. The main sources of iatrogenic aluminum are now aluminum-containing phosphate binders and aluminum-containing antacids administered to uremic patients or those with gastric or duodenal ulcer. Among compounds of this class pharmaceutical formulations containing $AlPO_4$ or $Al(OH)_3$ have found widespread use. With normal renal and gi function endogenous aluminum is readily excreted in the urine and feces. Thus, physiological serum level of aluminum is 0.07 to 0.3 μmol/L. However, high tissue load of aluminum may be found in patients with chronic renal failure who are treated by dialysis fluids that contain aluminum, or are given Al-hydroxide gels to control high plasma level phosphate. Increased content of aluminum may also appear in patients taking large quantities of Al-based antacids. Patients with high tissue and serum level of aluminum may develop blood, bone or brain diseases which may be linked to the excess of aluminum [1].

Many substances, such as dietary acids, may enhance the absorption of aluminum in healthy or sick persons by changing metabolic pathways of aluminum in gi tract. The substances that complex Al into stable, neutral complexes may ameliorate its absorption through gi membrane by increasing the lipid solubility of these complexes [2].

In gi tract, serum or tissue cells, pool of amino acids is present and their concentration may be, immediately after ingestion of protein rich food, very high. They may compete with other low-molecular-weight components for Al and affect its speciation [3]. Therefore, to fully characterize speciation of Al in body fluids and tissues it is necessary to study the interactions between Al and protein building α-amino acids.

So far comparatively small number of papers have been devoted to characterization of solution equilibria involving aluminum ion and α-amino acids and structure of the complexes, as well. Aluminum complexes with amino acids are generally weak, bearing in mind that $pK_a$ values of carboxyl group of the most amino acids is 2 – 3. In addition, the complex formation is considerably disturbed by pronounced hydrolysis of aluminum. Slow formation of various hydrolytic polymers of aluminum makes the determination of identity and stability of relatively weak complexes with amino acids, very difficult. Therefore, to observe the binary complex formation between aluminum and amino acids by potentiometric technique, high concentration ratios of amino acid to aluminum is required with concentration of the acid in the range 20 – 50 mmol/L and ligand to metal concentration ratio higher than 20. Since under these conditions strong buffering effect of amino acid prevents obtaining of reliable data, very precise measurements of hydrogen ion concentration, accurate data of aluminum hydrolysis, covering of as wide range of concentration ratios as possible, and strict control of experimental conditions are necessary.

Review of available literature data shows that no unambiguous description of aluminum complexation with any of α-amino acid exists [4-11]. Duc et al. [12] studied the complexation of aluminum with α-amino acids which constitute the collagen by potentiometric measurements in 0.5 mol/L $NaClO_4$ ionic medium at 298 K. They concluded that aluminum does not form complexes with glycine, alanine, serine, threonine, proline and hydroxyproline. The binary complex formation was found only in Al-Glu system while with histidine hydroxo mixed complexes were identified. The binary complexes of aluminum with number of amino acids (Gly, Ser, Thr, Asp, Glu and His) were characterized by Dayde and Berthon [13] in 0.15 mol/L NaCl ionic medium at $37^0$C. The log stability constants of the binary complexes were between 5.71 and 7.77. For the Al(His) complex the log stability constant was 7.08. Bearing in mind that complexes with histidine were studied in only two works and these with tyrosine were not studied at all, in the present paper we aimed to characterize the solution equilibria of aluminum with these two amino acids.

Histidine is involved in a large number of biochemical processes such as biosynthesis of histamine, secretion of prolactin and antidiuretic hormone, production of red and white blood cells, etc. Histidine possesses vasodilatating and hypotensive actions and may boost the activity of soothing alpha waves in the brain. Histidine is now used in the treatment of anemia, allergies, rheumatoid arthritis and other inflammatory reactions [14].

Tyrosine is synthesized in the body from phenylalanine and it is a direct precursor of adrenaline and thyroid hormones. Metabolic transformations of tyrosine require the presence of folic acid, niacin, vitamin C and copper. Its metabolic products include melanin, estrogen and encephalin. Tyrosine is used along with tryptophan to aid in the treatment of cocaine abuse and may also be useful in the control of anxiety or depression [15].

Therapeutic amounts of aluminum may bound to histidine and tyrosine and thus disrupt their metabolic pathways. Normal function of these amino acids may be thus, disturbed. In what extent such disturbance may occur, whether the Al-histidine or –tyrosine species are lipid soluble or not, can be answered if identity and stability of the complexes of Al with these two amino acids are reliably determined.

In this work we studied the complex formation between aluminum and histidine or tyrosine by using potentiometric, H-NMR and uv spectrophotometric measurements. Owing to pronounced hydrolysis in the studied systems, in the data treatment the hydrolytic scheme consisting of the complexes, $Al(OH)^{2+}$ (-5.27), $Al(OH)_3aq$ (-14.68), $Al(OH)_4^-$ (-23.0), $Al_3(OH)_4^{5+}$ (-13.81), $Al_{13}(OH)_{32}^{7+}$ (-109.23) and $Al(OH)_3(s)$ (-10.38) was used. The data were taken from our and the literature previous works [4,11,16].

### Experimental

*Reagents and Analysis*

The stock solution of aluminum(III) chloride was prepared by dissolving doubly recrystallized salt $AlCl_3$ 6 $H_2O$ p.a. (Merck) in twice distilled water. The appropriate amount of HCl was added to avoid initial hydrolysis of aluminum. The aluminum content was determined gravimetrically by the precipitation with 8-hydroxyquinoline and ammonia. Both methods gave the same results within 0.3%. The concentration of the free acid was determined potentiometrically using the Gran plot. The constancy of the total proton concentration in pure $AlCl_3$ solution with time was considered as a criterion for the absence of initial aluminum(III) hydrolysis and was periodically checked by titration against standard NaOH before each series of measurements.

L-histidine acid p.a (Sigma) and L-tyrosine p.a (Sigma) were dissolved in doubly distilled water and standardized by titration against standard NaOH.

Sodium hydroxide solution was prepared from concentrated volumetric solutions p.a. (Merck) by diluting with freshly boiled doubly distilled water, cooled under constant flow of purified nitrogen. The alkali concentration was checked by titration against potassium hydrogen phthalate. Hydrochloric acid solution was made from HCl "Suprapure" (Merck) and standardized against tris(hydroxymethyl) aminomethane. The solution of lithium chloride was prepared form LiCl, p.a (Merck) by dissolving re-crystallized salt in twice de-ionized water. The concentration was determined by evaporation of a known volume of solution to dryness at 573 K and weighing the residue.

*Equipment*

Potentiometric measurements were carried out using a Tacussel Isis 20000 digital pH-meter with a resolution ± 0.1 mV (in some measurements extended scale was used with a resolution ± 0.01 mV). The pH meter was equipped with a Tacussel TC-100 combined electrode. Titrant was delivered from a Metrohm Dosimat model 665. The constant temperature was maintained with VEB Prufgerate model E3E circulating ultrathermostat. H-NMR spectra of histidine and histidine + aluminum(III) ion solutions in $D_2O$, were recorded on Brucker WP 200SY spectrometer operating at 500 MHz. The pH of the histidine and mixtures of $AlCl_3$ and histidine in the required molar ratio was adjusted with KOD or DCl as necessary. The Al-His solutions tightly closed in a glass vial were heated to $40^0$C for 30 min and cooled to $25^0$C before the measuring the equilibrium pH. The pD values were calculated through the equation pD = pH + 0.4. For $^1$H-NMR measurements each solution was placed in a glass tube 5 mm in diameter. The rotation speed was set to

20 Hz. A $D_2O$ solution of 4,4-dimethyl-4-silapentane-1-sulfonate (DSS) enclosed in a glass tube 2.5 mm in diameter was used as an external standard. The chemical shifts were relative to TMS by using DSS as a secondary reference. Operating parameters were: td (number of points at which the FID is sampled): 16384, sw (spectral width): 8 - 20 ppm (4006 - 10000 Hz), p1(duration of the $^1H$ transmitter pulse): 3 μs, flip angle: $30^0$, d1 (relaxation delay): 1.0 s, ns (number of scans): 8. UV spectra of tyrosine and tyrosine + aluminum(III) ion solutions were taken on Varian SuperScan 3 uv-vis spectrophotometer. Matching pair of quartz cells with optical pathlength 1.0 cm were used. The 0.1 mol/dm$^3$ LiCl solution was used as reference blank.

*Procedure for potentiometric titrations*

All titrations were performed in a double mantled, thermostated glass vessel closed with the Teflon cork. The constant temperature, to (298.0 ± 0.1) K was maintained by circulating the thermostated water through the jacket. Purified and oxygen free nitrogen gas was bubbled through the solution for providing an inert atmosphere and stirring. Additional stirring of solution was achieved with magnetic stirrer.

The electrochemical cell used for potentiometric measurements may be represented as: *RE*/test solution (*TS*)/*GE* where *RE* and *GE* denote reference and glass electrode respectively. The general composition of test solution was: $TS = M$ $Al^{3+}$, $H$ $H^+$, $L$ aa (aa = $His^-$, or $Tyr^-$), 0.1 mol/dm$^3$ $Cl^-$, where $M$, $H$ and $L$ denote total molar concentrations of corresponding species.

Potential of the glass electrode is given by the expression $E = E_0 + Q \log h + E_j$, where $h$ is the concentration of free proton, $E_0$ is a constant which includes the standard potential of the glass electrode, $Q$ is the slope of the glass electrode response and $E_j$ is a liquid junction potential whose contribution to $E$ was found to be negligible. The $E_0$ was determined both before and during each titration of the test solution. First, $E_0$ was determined by means of separate titration of HCl with sodium hydroxide, both of known concentrations, under the same medium and temperature conditions as the test solution titrations (1.0, 2.5 and 5.0 mmol/L HCl was titrated with 0.100 mol/L NaOH). The data so obtained were analyzed with the aid of the Magec [17] program. The calculated values were $Q$ = 59.0 mV and self-protolysis constant of water, $pK_w$ = 13.75(2). During the titrations of the test solutions, the $E_0$ was determined using the data in acidic region, where no hydrolysis or complexation takes place ($h = H$), by plotting the value $E - Q \log h$ against $h$ and extrapolating the straight line so obtained to $h = 0$. When the difference between two $E_0$ values was higher than 2.0 mV, the titration was rejected. Thus, the obtained value of $E_0$ was used for the calculation of $-\log h$ for the whole titration curve.

To reduce the concentration of the hydrogen ion, the titrant was added stepwise in small aliquots (0.005 - 0.01 mL). The titrant was added, under energetic stirring of titrated solution. In this way initial formation of insoluble or colloidal aluminium hydroxide, which subsequently, dissolves very slowly, was avoided. Presence of colloidal aluminium hydroxide, at *p*H values around 5, was difficult to observe. The indication that titrated solution did not become supersaturated with respect to $Al(OH)_3$ were stable potential readings over prolonged period of time (we arbitrarily chosen to monitor the potential, at the end of titration, for additional 3-4 hr.). The potential was monitored after each addition of a titrant. The readings were taken every 2 min until steady values to ± 0.1 mV/min were obtained. Usually stable potential readings were obtained in 5 – 10 min after the addition of the titrant. If in the specified time interval no stable potential reading was obtained the corresponding point was excluded from calculation. The titrations were terminated when drifted potential readings were obtained and turbidity of solutions observed. Some titrations were carried in duplicate and some in triplicate.

*Data Treatment*

Three kinds of equilibria should be considered in the present study: (a) protonation of histidine and tyrosine anions, (b) hydrolysis of aluminum(III) ion, and (c) general three component equilibria,

$$p\,Al^{3+} + q\,H^+ + r\,aa \rightleftharpoons [Al_pH_q(aa)_r]^{(3p+q-r)+};\ _{p,q,r}$$

which include the case $q$ =0, i.e. the formation of pure amino acid complexes of $Al^{3+}$. Negative values of $q$ represent hydroxo complexes. The overall protonation constants of amino acid ions and stability constants of hydrolytic complexes of aluminum(III) ions were determined in separate experiments. Thus, in evaluation of three component equilibria (c), the binary models (a) and (b) were considered as known.

The mathematical analysis of the experimental data was performed with the aid of general least-squares program Superquad [18]. In Superquad calculations the identity and stability of complexes which give the best fit to the experimental data, were determined by minimizing the error-squares sum of the potentials, $U$: $U = \Sigma w_i (E_{obs} - E_{calc})^2$

where $w_i$ represents a statistical weight assigned to each point of titration curve, $E_{obs}$ and $E_{calc}$ refer to the measured potential of the cell and the calculated one assuming the specific model and trial constants, respectively. The best model was chosen using these criteria: (a) the lowest value of $U$, (b) standard deviation in calculated stability constants less than 0.15 log units, (c) standard deviations in potential residuals, defined as:

$$s = \{ewe^T / (N-k)\}^{1/2}$$

where $e$ is a vector in potential residuals ($E_{obs}$ - $E_{calc}$), $w$ is a weighting matrix, $N$ is the number of observations and $k$ is the number of refinable parameters, with standard deviation in volume readings 0.0005 cm$^3$ and standard deviation in potential readings 0.1 mV, should be less than 3.0. (d) goodness-of-fit statistics, $\beta^2$ (Pearson's test) at 95% confidence level, with 6 degree of freedom, less than 12.6 and (e) reasonably random scatter of potential residuals without any significant systematic trends. Along with Superquad the program Best [19] was also used in calculations. All calculations were performed on a PC IBM PIII/800 compatible computer.

### Results and discussion

*Protolytic equilibria in pure amino acid solutions*

Overall protonation constants, $\beta_i$ of histidine and tyrosine defined as:

$$i\,H^{+} + aa^{-} \rightleftharpoons Haa^{(i-1)+}, \qquad \beta_i$$

were determined from glass electrode potentiometric titration data of the solutions of the corresponding amino acids. Three total concentrations of histidine, 1.0, 1.5 and 2.0 mmol/L were used and 1.0 and 2.0 mmol/L of tyrosine. Appropriate amount of standard HCl was added in each solution. In total 300 points were collected for histidine and 250 for tyrosine. The constants were calculated with the aid of Superquad program. The obtained values for histidine: log $\beta_1$ = 9.17 ± 0.02; log $\beta_2$ = 15.29 ± 0.03; log $\beta_3$ = 17.01 ± 0.06 and tyrosine: log $\beta_1$ = 10.07 ± 0.01; log $\beta_2$ = 19.18 ± 0.04 and log $\beta_3$ = 21.38 ± 0.09 are in good agreement with literature data [20]. It should be noted that dissociation of phenolic –OH group of tyrosine was taken into account in pure protonation calculation. However, in subsequent calculations relating to complex formation, no dissociation of –OH group was considered, i.e. only protonation of amino and carboxylate groups were considered.

*Equilibria in aluminium(III)+histidine solutions*

*Potentiometric measurements*

A summary of the potentiometric experimental data is given in Table 1.

**Table 1.** Summary of potentiometric data obtained in $Al^{3+}$- His and $Al^{3+}$ - Tyr systems in 0.1 mol/L LiCl ionic medium at 298 K. All total concentrations, $C_X$ are in mmo/L. The maximum attained average ligand number is denoted as $Z_c$.

| Run no. | $C_{Al}$ | $C_{His}$ | $C_{Tyr}$ | $C_{HCl}$ | pH interval | $Z_c$ |
|---|---|---|---|---|---|---|
| 1 | - | 1.0 | - | 17.10 | 1.885-10.177 | - |
| 2 | - | 1.5 | - | 17.50 | 1.870-10.150 | - |
| 3 | - | 2.0 | - | 18.06 | 1.865-10.152 | - |
| 4 | 0.5 | 1.0 | - | 4.07 | 2.614-6.414 | 1.74 |
| 5 | 1.0 | 2.0 | - | 5.50 | 2.527-6.087 | 1.89 |
| 6 | 2.0 | 4.0 | - | 7.53 | 2.564-5.798 | 1.67 |
| 7 | 0.5 | 1.5 | - | 11.96 | 2.048-6.425 | 1.68 |
| 8 | 1.0 | 3.0 | - | 7.06 | 2.510-6.270 | 1.88 |
| 9 | 2.0 | 6.0 | - | 10.05 | 2.519-5.619 | 1.59 |
| 10 | 0.5 | 2.5 | - | 5.98 | 2.554-6.518 | 1.95 |
| 11 | 1.0 | 5.0 | - | 8.49 | 2.616-6.271 | 1.91 |
| 12 | 2.0 | 10.0 | - | 13.99 | 2.599-6.088 | 1.82 |
| 13 | - | - | 1.0 | 11.96 | 2.011-10.440 | - |
| 14 | - | - | 2.0 | 11.96 | 2.041-10.058 | - |
| 15 | 0.5 | - | 1.0 | 11.96 | 1.994-6.974 | 2.40 |
| 16 | 1.0 | - | 2.0 | 11.96 | 2.029-6.708 | 2.70 |
| 17 | 1.5 | - | 3.0 | 11.96 | 2.056-6.887 | 2.34 |
| 18 | 0.5 | - | 1.5 | 11.96 | 2.016-6.013 | 2.90 |
| 19 | 1.0 | - | 3.0 | 11.96 | 2.020-6.774 | 2.77 |

In the pH range studied (2.5 – 6.5) the maximum apparent ligand number reached was ca. 1.9. The highest concentration ratio of histidine to $Al^{3+}$ was 5:1. Beyond pH 6.2, the solutions became turbid and drifting potential readings were obtained. No higher concentration ratios of histidine to Al were used because they would seriously change the constancy of the medium. In addition, the buffering effect of histidine may hinder the reliable potentiometric measurements. The establishing of equilibrium in solutions was moderately slow especially at the pH values higher than 4.5. High turbidity of solutions was observed at pH values near 6.5.

In order to derive the speciation model for the studied system the experimental data were plotted as the dependence of average ligand number, $Z_{Al}$ on *–log[His]*.

Average ligand number of aluminium was calculated through the formula:

$$Z_{Al} = \frac{C_{His} - [His]\{1 + \sum_n \beta_n [H^+]^n\}}{C_{Al}}$$

where $C_X$ denotes total analytical concentration of the species $X$, while $[X]$ stands for equilibrium concentration of $X$. The equilibrium concentration of histidine was calculated using the formula:

$$[His] = \frac{C_H - [H^+] + [OH^-]}{\sum_n n\beta_n [H^+]^n}$$

where symbols have their usual meaning.

Analysis of formation curves, (Fig 1), provides information that there are more species then simply Al(His) and/or $Al(His)_2$. Systematic dependence of the formation curves on total metal and total ligand concentrations indicate the presence of ternary complexes.

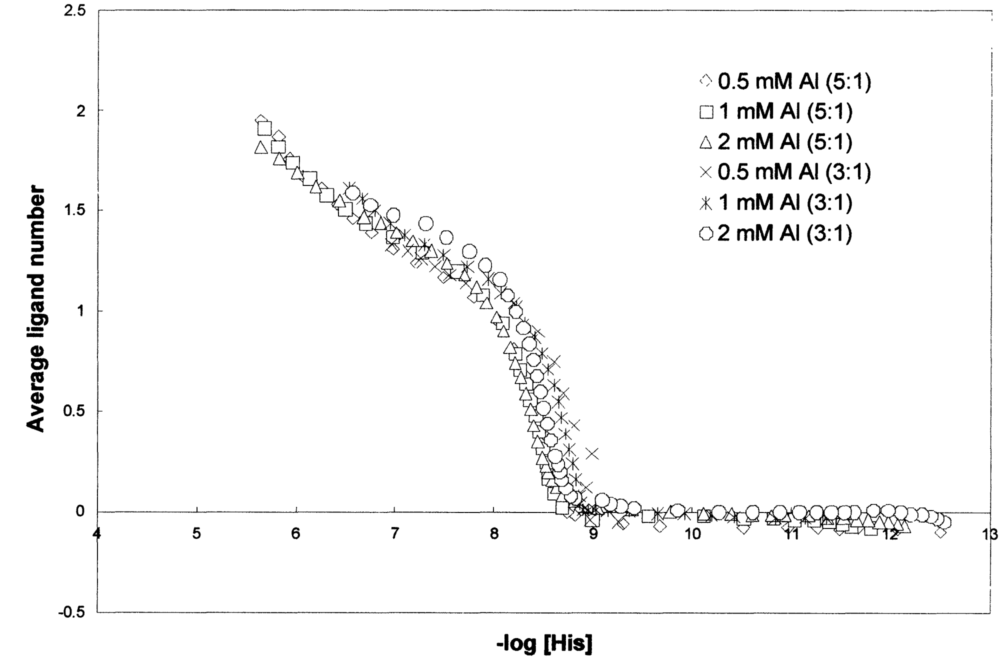

Figure 1. Formation curves in $Al^{3+}$ + L-Histidine solutions obtained by glass electrode potentiometric measurements at 298 K in 0.1 mol/L LiCl ionic medium.

For p[His] > 9 all formation curves superimpose. At p[His] ~ 9 curves begin to spread. This is where the concentration of Al(HHis) maximizes. The maximum spread is reached at p[His] ~8 where both Al(HHis) and Al(HHis)(His) are present. When Al(His) predominates the formation curves begin to superimpose once again

The equilibria in the His + $Al^{3+}$ system may be represented in a general form:

$$p\,Al^{3+} + q\,H^+ + r\,His^- \rightleftharpoons Al_pH_q(His)_r$$

The stability constants of various (p,q,r) species formed in the above reaction, may be defined as:

$$\beta_{p,q,r} = C_{p,q,r}\, m^{-p} h^{-q} l^{-r}$$

where $C_{p,q,r}$ denotes the equilibrium concentration of the complex, $m$, $h$ and $l$ denote free concentrations of aluminium(III), proton and histidine, respectively. To determine the composition and stability constants of the species formed, the titration data were analyzed using the programs Best and Superquad. The following complexes were selected to find the model which best fit the experimental data: (1,0,1), (1,0,2), (1,1,1), (1,2,1), (1,1,2), (1,-1,1), (1,-2,1), (1,-3,1), (1,-1,2), (1,-2,2), (1,-2,3) as well as some polymers. More than 20 various models were tested. The stability constants of the hydrolytic complexes of aluminium and protonated species of histidine were not refined during the calculations. First, each titration curve was treated separately using the program Best. Complexes were added in the model one at a time until the lowest value of $\sigma_{fit}$ was achieved (usually less than 0.003). These complexes were then used as the starting model for the Superquad calculations. Then the data belonging to all titration curves, referred to one particular histidine to aluminium concentration ratio were treated together. The refined values of $E_0$ served as the additional criterion for model selection. If they were different from experimental ones for more than 0.5 mV, the model was considered as inadequate. The finally accepted set of complexes is given in Table 2.

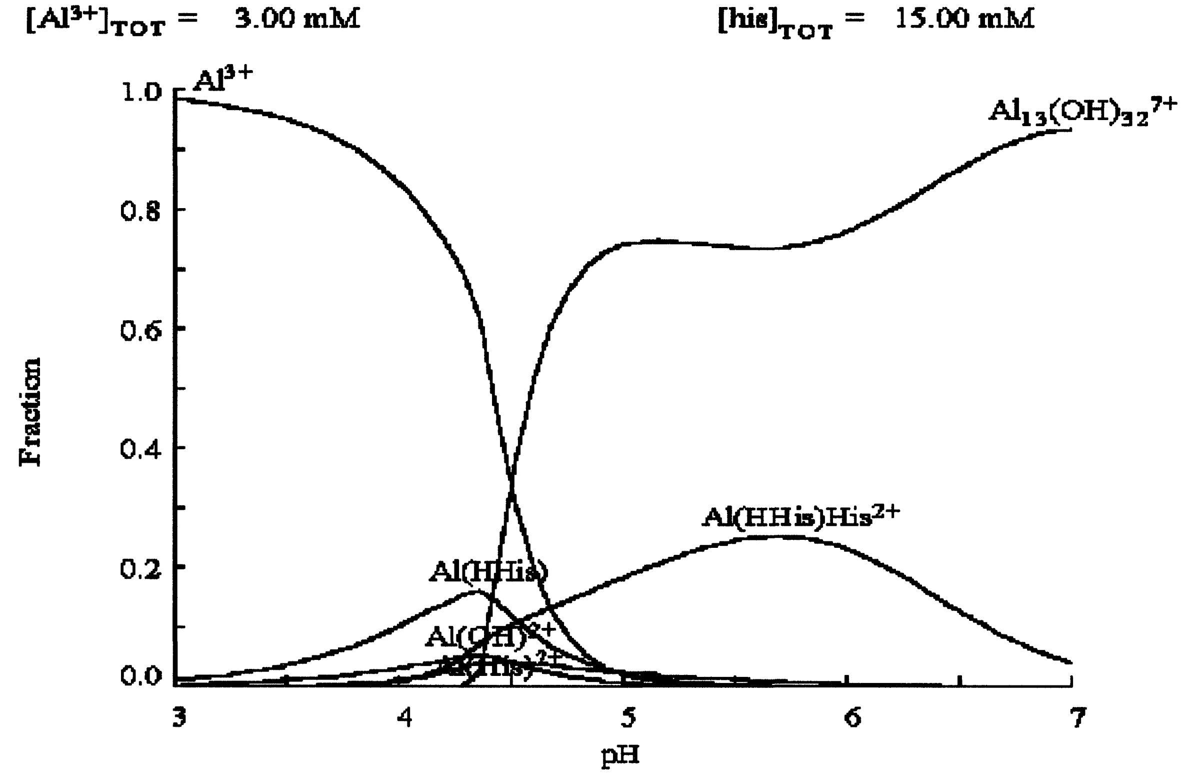

Figure 2. Distribution of species in $Al^{3+}$ + L-Histidine solution

The distribution diagram of Al-histidine species is shown in Fig. 2. At total Al concentration 1.0 mmol/L and ligand to metal ratio 5:1 maximum formation of $Al(HHis)^{3+}$ complex takes place at the pH 4.5. In parallel with this complex the complex Al(His) is also formed. The distribution diagram indicates that in the pH interval 4.0 – 4.5 most important reactive species of aluminium is monohydroxo complex $Al(OH)^{2+}$ so that the probable mechanism of the formation of binary ligand complexes should be:

$$Al(OH)^{2+} + H_2His^{+} \rightleftharpoons Al(HHis)^{3+} + H_2O$$

$$Al(OH)^{2+} + HHis \rightleftharpoons Al(His)^{2+} + H_2O$$

Bearing in mind the concentrations of the reactive species in the pH region in which Al(His) complex is formed, it seems reasonable to suppose that the actual composition of the complex is Al(OH)(HHis), i.e. that one of coordinated water molecules to aluminum is protolyzed and ammonium group of histidine does not dissociate. Protolysis is facilitated by the vicinity of imidazole nucleus and possible formation of hydrogen bonding with imidazole nitrogen.

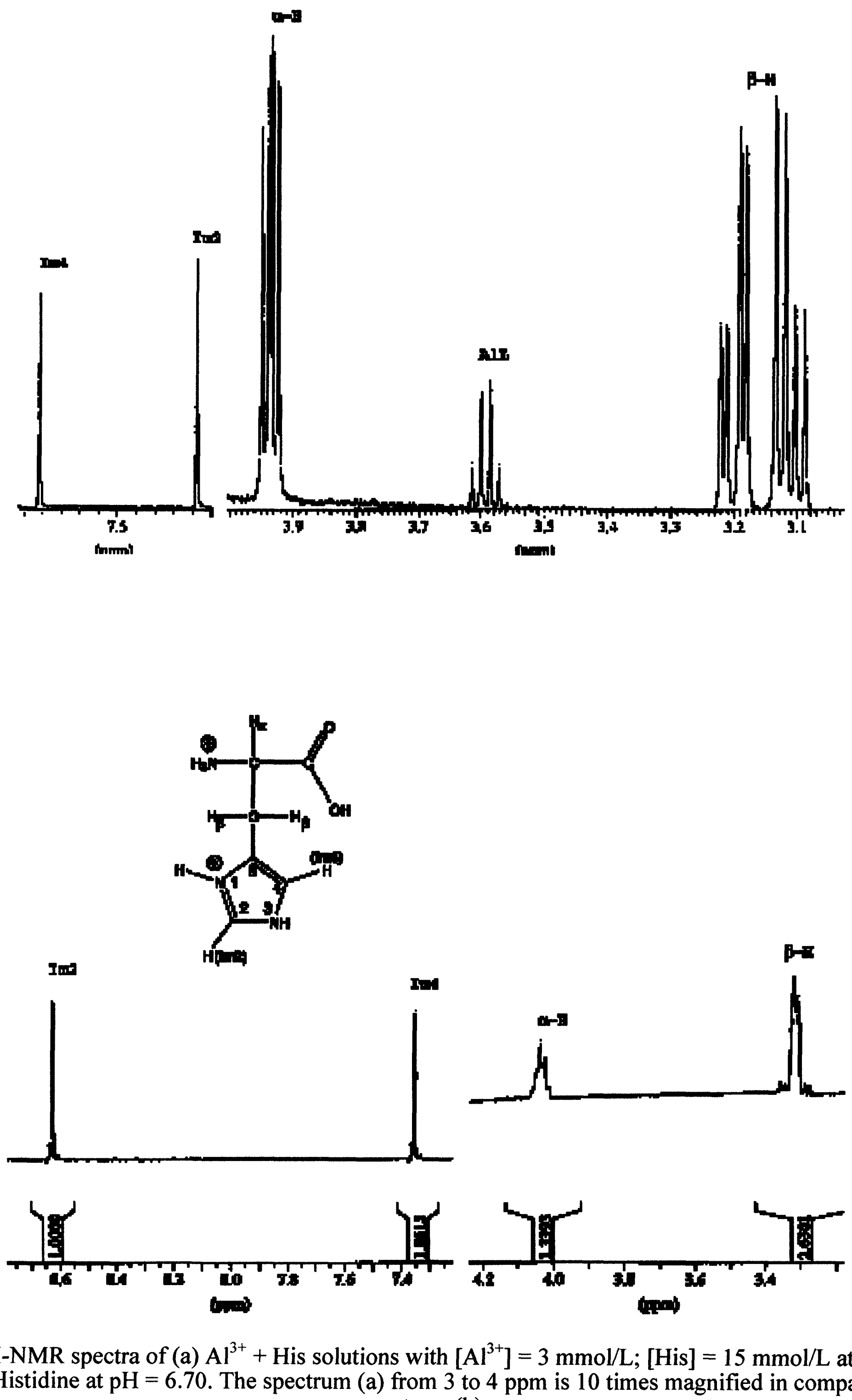

Figure 3. H-NMR spectra of (a) $Al^{3+}$ + His solutions with $[Al^{3+}]$ = 3 mmol/L; [His] = 15 mmol/L at pH = 6.70 and (b) L-Histidine at pH = 6.70. The spectrum (a) from 3 to 4 ppm is 10 times magnified in comparison with spectrum (b).

**Table 2.** Calculated stability constants of $Al^{3+}$ - His and $Al^{3+}$ - Tyr complexes in 0.1 mol/L LiCl ionic medium at 298 K. Overall stability constants were defined as: $\beta_{p,q,e} = C_{p,q,r}\ [Al]^p[H]^q[aa]^r$ where p,q,r denote stoichiometric indices and aa stands for histidine or tyrosine.

| Species | Log $\beta_{p,q,r} \pm \sigma$ | | Literature value |
|---|---|---|---|
| | Potentiometry | Spectrophotometry | |
| Al(HHis) | 12.21 ± 0.08 | - | - |
| Al(HHis)His | 20.3 ± 0.1 | - | - |
| Al(His) | 7.25 ± 0.08 | - | 7.08 ± 0.20 [15]<br>8.03 ± 0.10 [14] |
| Statistics | $\chi^2$ =10.7-12.0; s = 2.0-3.5 | - | |
| Al(HTyr) | 12.72 ± 0.09 | 11.94 ± 0.10 | |
| Al(Tyr) | 10.16 ± 0.03 | 9.71 ± 0.08 | |
| $Al(OH)_2Tyr$ | - | 2.70 ± 0.05 | |
| Statistics | $\chi^2$ = 10 – 12; s = 2 –3 | SD = 0.005; S = 0,01 | |

If however, the formation of aqueous and solid $Al(OH)_3$ is taken into account then the formation of mixed bis complex Al(HHis)His becomes significant. It probably forms by stepwise mechanism:

$$Al(His)^{2+} + HHis \rightleftharpoons Al(HHis)His^{2+}$$

*Proton magnetic resonance measurements*

H-NMR measurements were made on solutions of histidine and $Al^{3+}$ + histidine with total metal concentration 3 mmol/L and ligand to metal concentration ratios 2:1, 3:1, 5:1 and 10:1 at pH values 3.53; 4.50; 5.45; and 6.72. The proton NMR spectrum of histidine consists of proton resonances of hydrogen atoms bonded to carbon atoms designated as α, β, Im2 and Im4 [21]. The other protons of histidine exchange rapidly with water. The pH dependencies of the chemical shifts of the four types of non-exchanging hydrogen atoms of histidine that the resonances of α-H and β-H respond to the smallest degree to all of the proton ionizations while the resonances of Im2-H considerably shift toward lower field (from 8.0 to 8.63 ppm) in going form pH 3.52 to 6.75. This is certainly due to imidazole 1-N proton ionization in this pH range. In $Al^{3+}$ + His spectra large shifts of β-H and Im2 proton resonances of histidine hydrogen atoms, upon increasing the pH, in comparison with histidine resonances in absence of aluminum, were observed. The α-H and Im4 resonances show considerably smaller shifts under these conditions. The spectra of histidine and $Al^{3+}$ + His solutions at total aluminum concentration 3.0 $mmol/dm^3$ and total histidine concentration 15.0 $mmol/dm^3$ at pH 6.75 are shown in Fig. 3. As can be seen from Fig. 3 all proton resonances are shifted to higher fields in comparison with uncomplexed histidine.

In the pH range 3 – 5 proton resonances in $Al^{3+}$ + His solutions are shifted to lower fields in comparison with these of histidine in the absence of aluminum. However, at pH 6.75 new resonances, not seen in the spectrum of pure histidine, are observed at 3.57, 3.58, 3.60 and 3.61 ppm. These resonances increase in intensity upon rising the concentration ratio of histidine to aluminum up to 10 : 1. The observed shifts of proton resonances and appearance of new resonances in Al + His solutions indicate complex formation in these solutions. It was not possible to deduce the stoichiometry of the complexes formed due to parallel formation of several complexes. The splitting and shifts of α-H and β-H resonances as well as their broadening indicate coordination of the carboxyl group of histidine to aluminum. Aliphatic nitrogen in the pH range 3 – 7 is not bound to aluminum. Though it can not be on the basis of available data, unquestionably concluded whether aromatic imidazole nitrogen is coordinated to aluminum or not, it seems that Im1 nitrogen may take part in coordination. It is supported by the fact that Im2 proton resonance at pH between 3 and 5, shifts from 8.0 to 8.62 ppm and at pH 6.75 from 8.63 to 7.92 ppm upon coordination of histidine. No broadening or splitting of this resonance was observed. Thus, the Al – His complex probably exists in both, monodentate and bidentate forms (Scheme 1). Further multinuclear nmr studies are necessary to confirm or decline this assumption. Thus, the structures proposed in Scheme 1 are only tentative.

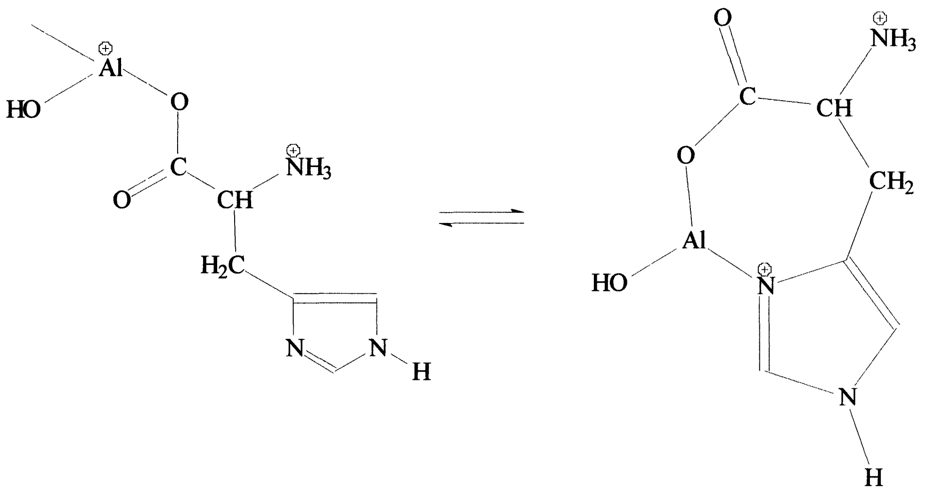

Scheme 1. Possible structure of Al(His) complex

*Molecular modeling*

To see which of two probable structures is dominating molecular mechanics calculations were done with HyperChem release 6.0 professional version [22], an interactive graphics program that allows rapid structure building, geometry optimization and molecular display. Energy minimization was repeated several times to find the global minimum. From molecular mechanics (MM+) it was found that the minimized energy for linear (monodentate) structure (34.98 kcal/mol) is lower than that of the cyclic (bidentate) one (39.12 kcal/mol). Therefore, linear structure is preferred.

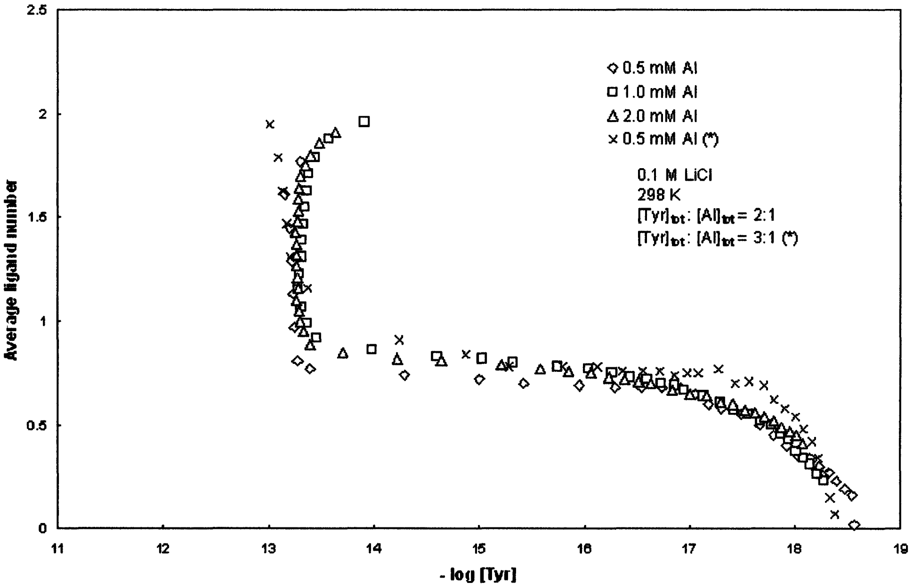

Figure 4. Formation curves in $Al^{3+}$ + L-Tyrosine solutions obtained by glass electrode potentiometric measurements at 298 K in 0.1 mol/L LiCl ionic medium.

*Equilibria in aluminium(III) + L-Tyrosine solutions*

*Potentiometric measurements*

Summary of the potentiometric experimental data is given in Table 1. Owing to small solubility of tyrosine maximum ligand to metal concentration ratio achieved was 3:1. The titrations were commenced at rather low pH values ca. 2.0, and extended up to pH ca. 7.0. To avoid the precipitation region starting at pH 6, two sets of titrations were performed. In first set the titrations were carried out up to pH 4.5 using the protocol described in Experimental section. In second set of titrations the pH of solutions was brought up to pH ca. 4.0 with very slow addition of alkali and solutions were left overnight. Next day the pH of solutions was re-checked and titrations were commenced with the addition of very small aliquots of alkali (0.005 mL) in 2 min time intervals. The solutions stayed clear up to pH 6.5 when plenty of precipitation formed. The formation curves *Z = f(-log[Tyr])* are shown in Fig. 4.

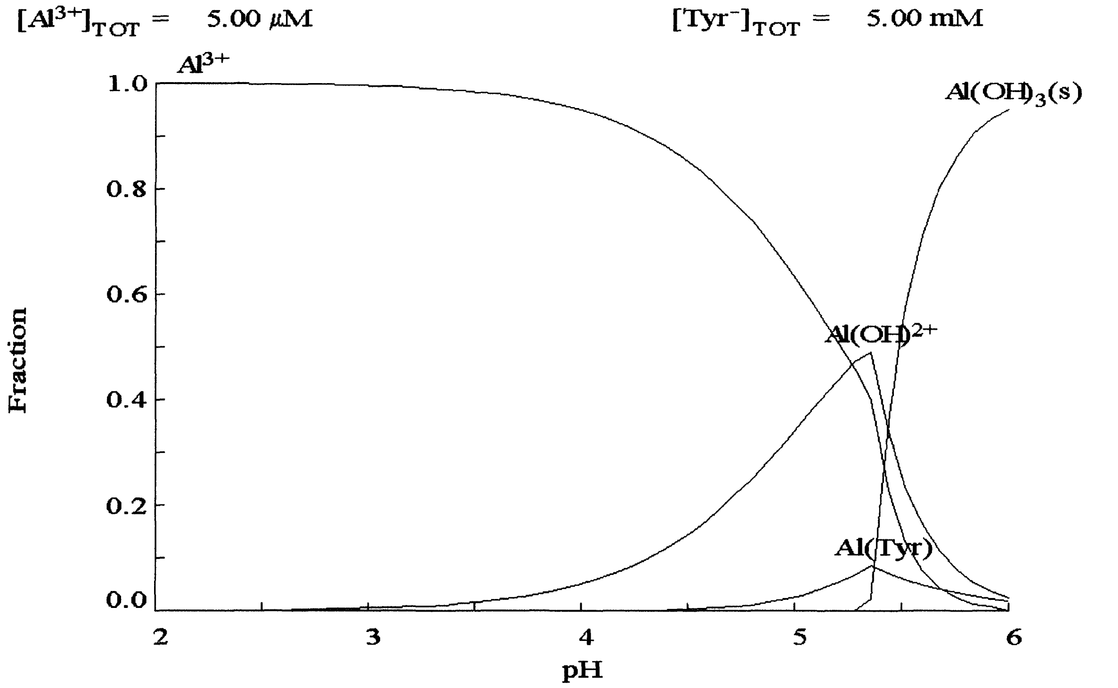

Figure 5. Distribution of species in $Al^{3+}$ + L-Tyrosine solution

It can be seen that up to pH 6.0 only mononuclear complexes are formed while beyond this value extensive hydrolysis dominates in all solutions. For the calculation purpose the number of data points was reduced and pH intervals 2.0 – 4.5 and 4.5 to 6.5 were treated separately. In the mathematical treatment of the first set of titration curves protonation constants of tyrosine and hydrolytic stability constants of aluminum were held fixed while varying the trial stability constants of the Al – Tyr complexes. The only accepted complex was $Al(HTyr)^{3+}$ with a stability constant, log $\beta_{1,1,1}$ = 12.72 ± 0.09 and reasonably good set of statistics, $\chi^2$ = 11.9 and s = 3.0. Attempts to fit the data with binary complex Al(Tyr) were unsuccessful. Mathematical treatment of the second set of titrations indicated that no one combination of mononuclear complexes can give acceptable fit. Since in this pH region main hydrolytic complexes are (1,-3), (13,-32), (1,-4) and (3,-4) their stability constants were allowed to vary. Two models were accepted. The first one consisted of mixed hydrolytic complexes $Al(OH)Tyr^{+}$ (log $\beta_{1,-1,1}$ = 7.15 ± 0.03) and $Al(OH)_2Tyr$ (log$\beta_{1,-2,1}$ = 2.70 ± 0.05) with statistical parameters: $\chi^2$ = 10.1 and s = 2.9, and the other one consisting of $Al(Tyr)^{2+}$ (log $\beta_{1,0,1}$ = 10.8 ± 0.3 and $Al(OH)_2Tyr$ (log $\beta_{1,-2,1}$ = 2.74 ± 0.08) complexes with set of statistics $\chi^2$ = 11.1, s = 3.0. In the first set of complexes all the hydrolytic complexes except (1,-4) and (13,-32) were rejected, while in a second set only (3,-4) complex was rejected. Thus, it is obvious that hydrolysis obscures the complexation. To overcome this problem the new hydrolytic complex (1,-2) was introduced into the calculation and the stability constant of (13,-32) complex was fixed. The acceptable fit was obtained with the complexes Al(Tyr) and $Al(OH)_2Tyr$ with set of statistics, $\chi^2$ = 11.2, s = 3.0. The stability constants of the hydrolytic complexes changed for ca. 10% and the complex (1,-2) was accepted. Since the stability constant of the binary complex, $Al(Tyr)^{2+}$ had rather large standard deviation in the next calculation cycle data points were restricted to the pH interval 2.0 – 6.0 with fixed stability constant of Al(HTyr) complex and floating stability constants of the hydrolytic species. The calculation ended with the acceptance of the Al(Tyr) complex with the stability constant log $\beta_{1,1,0}$ = 10.16

± 0.03 and set of statistics, $\chi^2 = 12.0$ and s = 2.80. Scatter of residuals was reasonably random, thus making the obtained speciation model convincingly reliable. Therefore in the final speciation scheme including the complexes Al(HTyr), Al(Tyr) is given in Table 2.

The species distribution diagram (Fig 5) indicates that hydrolysis dominates over the entire investigated pH range. Taking the "therapeutic" concentrations of aluminum (5 µmol/L) and tyrosine (5 mmol/L) it can be seen that at such low concentration of aluminum, only mononuclear complexes are formed; the complex Al(Tyr) begins to form at pH values higher than 4.0. Total fraction of this complex is rather small and upon increasing the pH its concentration begins to decrease. At pH values higher than ~5.5 the whole system is micro-heterogeneous due to the formation of colloidal $Al(OH)_3$.

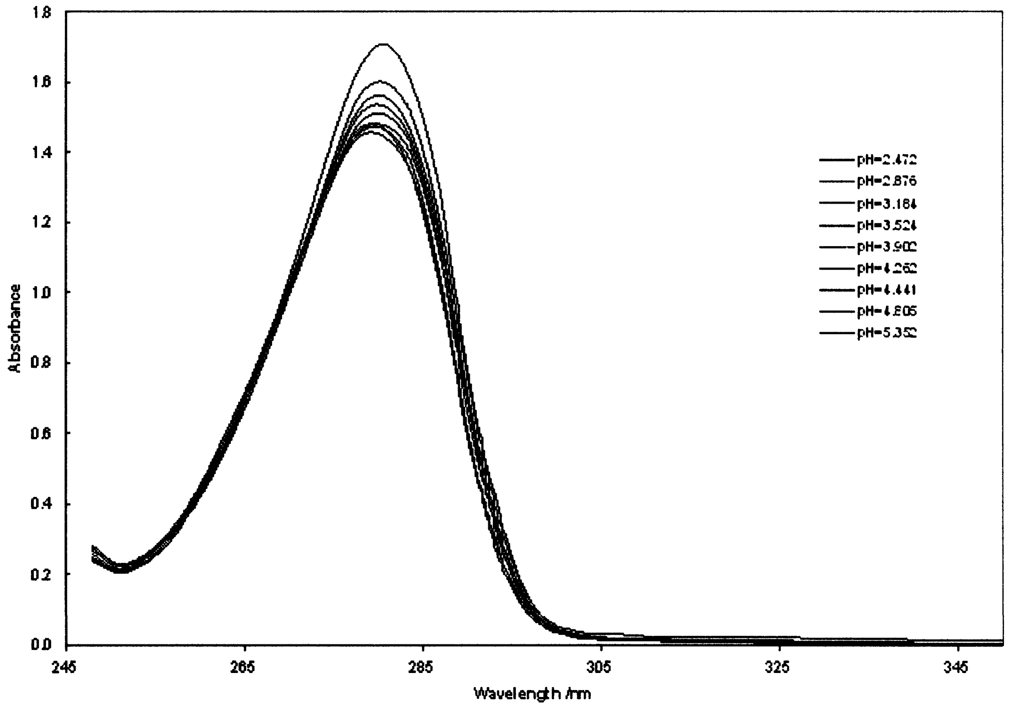

Figure 6. UV spectra of $Al^{3+}$ + L-Tyrosine solutions $[Al^{3+}]$=1.0 mmol/L; [Tyr]=5 mmol/L. The spectra are not corrected for tyrosine absorption.

*Spectrophotometric measurements*

To confirm the complexation in the Al – Tyr system and to improve the potentiometrically obtained speciation scheme, the uv spectra of Al – Tyr solutions were taken in a wide range of pH values and concentration ratios of ligand to metal. The tyrosine intensively absorbs in near – uv region and this property was used to follow the complexation in Al – Tyr solutions. The spectra of the tyrosine solutions, in the absence of aluminum, were taken in the pH interval from 1.90 to 10.50 for total tyrosine concentrations 0.5 and 1.0 mmol/L. In total 25 solutions were prepared. Larger number of solutions were prepared in the pH interval 8.70 to 10.50 owing to overlapping equilibria of phenolic and ammonium group dissociation. Spectrum of fully deprotonated tyrosine was obtained in 0.1 mol/L NaOH solution, while the spectrum of fully protonated tyrosine was obtained in 0.1 mol/L HCl solution. Large number of spectra and precise pH measurements (precision ± 0.002 pH units) allowed obtaining of the molar absorptivities of all intermediate tyrosine species i.e. di- and monoprotonated ones, together with corresponding thermodynamic equilibrium constants, with the aid of the program Squad [23]. Since however, our measurements of complexation was limited to pH about 6.0 where neither of groups phenolic or ammonium appreciably dissociate, we further considered only the species $Tyr^-$, $HTyr^{\pm}$ and $H_2Tyr^+$. Since phenolic –OH group of tyrosine does not dissociate in the pH range used for complexation measurements, there is no need to consider fully deprotonated tyrosine as a ligand; it is more convenient that the ligand $Tyr^-$ represents deprotonated tyrosine zwitterion. At the same time it means that only three micro-species, out of eight possible, should be taken into consideration. Macro-constants that correspond to these species involve dissociation of ammonium and carboxyl groups. The calculated values of the overall protonation constants log $\beta_1$ = 9.31 ± 0.02 and log $\beta_2$ = 11.70 ± 0.05 are in good agreement with the potentiometric data. ($\beta_1$ refers to protonation of amino group, $\beta_2$ refers to overall protonation of amino and carboxylate groups).

Spectrophotometric measurements on Al – Tyr solutions were made at total aluminum concentrations 0.25, 0.5 and 1.0 mmol/L and tyrosine to aluminum concentration ration 2:1 to 5:1. The pH of the solutions was varied from pH ca. 2.0 to ca. 5.5 with the appropriate addition of HCl and/or KOH. The spectral interval was from 245 to 345 nm. After preparation solutions were left overnight and the spectra were taken next day at every hour until no changes of absorbance higher than ± 0.02 were detected. The spectra obtained at tyrosine to aluminum concentration ratio of 5:1 are shown in Fig. 6.

The spectral bands are centered at 281 nm and show slight red shift and increase in intensity upon rising the pH. For the calculation purpose the spectra were digitized at every 2 nm. The identity and stability of the complexes formed were determined by minimizing the error-square sum of the absorbance:

$$S = \sum (A_{i,obs} - A_{i,calc})^2$$

where the calculated absorbency is given as:

$$A_{calc} = \sum_{p,q,r} \beta_{p,q,r} \varepsilon_{p,q,r} [Al]^p [H]^q [Tyr]^r$$

The minimization of the sum, $S$, was performed with the aid of the program Squad [23]. The same model consisting of the complexes Al(HTyr) and Al(Tyr) was accepted at all concentration ranges of tyrosine to aluminum. However, these complexes were accepted only after removal of the hydrolytic tridecamer, (13, -32). Other hydrolytic species were held fixed. At 2:1 concentration ratio improved fit was obtained after introduction of mixed hydrolytic complex $Al(OH)_2Tyr$. This complex was not accepted at concentration ratios 3:1 and 5:1. Also, polynuclear species were not accepted. The goodness of fit was judged by the calculated statistical parameters: SD – standard deviation in absorbance data and S, the sum of squares of the residuals. The fit was considered adequate if SD is less than 1 x $10^{-2}$ and S less than 1 x $10^{-1}$. The calculated statistical values were at 2:1 concentration ratio SD = 1.3 x $10^{-2}$ and S = 0.05; at 3:1 SD = 8 x $10^{-3}$ and S = 0.01 and at 5:1, SD = 5 x $10^{-3}$ and S = 0.01. Thus, the fit may be considered as satisfactory.

The calculated spectra of the Al(HTyr) and Al(Tyr) complexes are given in Fig. 7. As seen from fig. 7, the spectra are similar in shape, but maximum absorption coefficient is higher for the protonated complex.

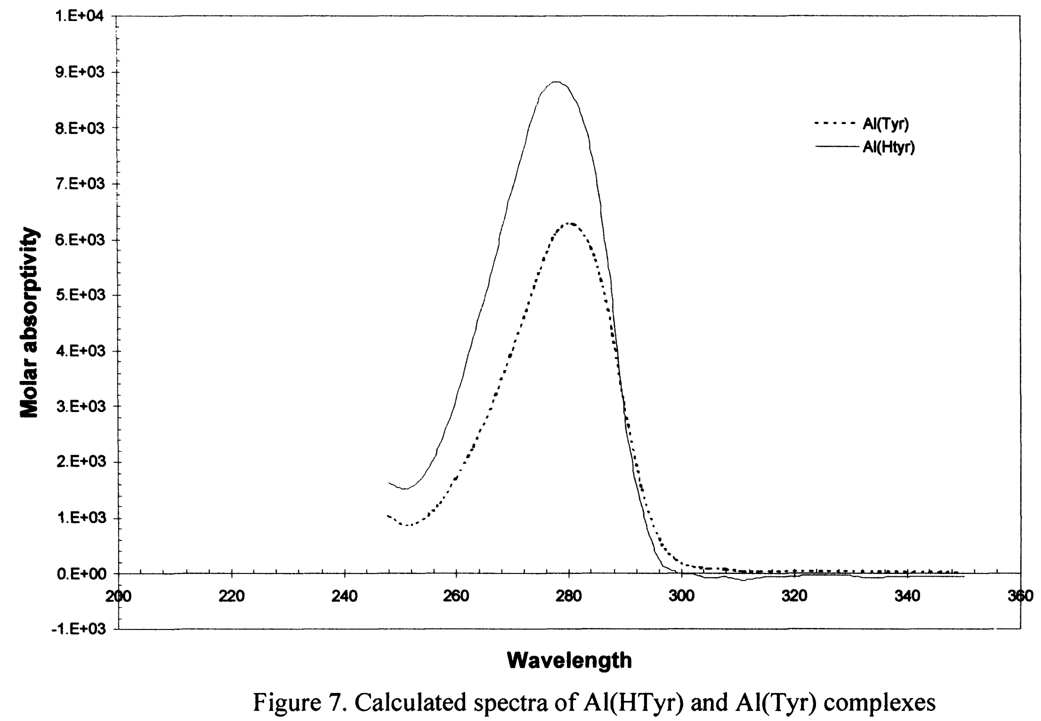

Figure 7. Calculated spectra of Al(HTyr) and Al(Tyr) complexes

Since the absorption band of tyrosine does not undergo significant change in shape and no new band is produced in the presence of aluminum it may be concluded that the ligation proceeds far from benzene. Thus, tyrosine acts as a monodentate ligand with carboxylate group bonded to aluminum. Amino nitrogen in Al(HTyr) is not involved in complexation, though dissociation of proton from ammonium group is facilitated upon coordination of aluminum to carboxylate group. Concerning the Al(Tyr) complex, similarly as in a case of Al(His) complex, its composition could be represented as Al(OH)(HTyr), i.e. it does not seem probable that ammonium group can dissociate in the pH region in which this complex is formed. On the other hand vicinity of large hydrophobic benzene group facilitates protolysis of coordinated water molecule so that mixed hydroxo complex is formed in solution.

The results of the present study indicate that in the title systems competition between hydrolytic and complexation equilibria exists, so that the transport and accumulation of aluminium must be considered in terms or these equilibria, taking place in body fluids. Equally significant role in addition to complexation, play kinetic factors. The dominance of amino acids – aluminium ion equilibria may be expected in tissues and compartments where the concentration of free amino acids is high, as well as their concentration ratio to aluminium. Such situation may arise in duodenum or in kidneys. Free histidine or tyrosine in kidney ultrafiltrate may complex aluminum and if charged, these complexes may enhance the excretion of Al. Bearing in mind slightly acidic pH in kidneys, one may expect the formation of mostly binary or mixed hydrolytic complexes between aluminium and histidine. Since they are all charged, excretion may be enhanced. However, aluminium may me also deposited in kidneys, depending on exposure to exogenous Al-compounds, age, physiological state of organism, etc. Its toxic effect is mostly pronounced on proximal tubules. In pathological cases, when tubular re-absorption of amino acids is greatly reduced (so that they reach about 10 to 20 times higher concentration in primary urine than physiological), formation of neutral hydroxo aluminium complexes may prevail. Consequently, mobilization of aluminium from kidney deposits may occur. The formation of aluminium complexes with zwitterionic forms of amino acids may also be a factor that increases aluminium toxicity. If high concentrations of histidine or tyrosine are ingested concomitantly with aluminium based antacids then, in pathologically altered gi membranes, absorption of aluminium may occur with consequent toxic effects.

**Acknowledgement:** The authors express their gratitude to Prof. Guy Berthon (Toulouse, France) for reading the ms and valuable suggestions and to Prof. Imre Toth (Debrecen, Hungary) for help with nmr measurements.

### References

1. C. Alfrey, Toxicity of detrimental metal ions: aluminum. In G. Berthon (editor), *Handbook of metal-ligand interactions in biological fluids. Bioinorganic medicine*. Vol. 2. Marcell Dekker, 1995. pp. 735-742; In G. Berthon, *Coord. Chem. Rev.*, **149** (1996) 241; G.A Taylor, P.B Moore, Ferrier, S.P Tyrer, J.A Edwardson, *J. Inorg. Biochem*. **69** (1998) 165
2. M. Venturini Soriano, G. Berthon, *J. Inorg. Biochem.*, **69** (1998) 1, N. Alliey, M. Venturini Soriano, G. Berthon, *Annals Clin. Lab. Sci.*, **26** (1996) 122, G. Berthon, S. Dayde, *J. Am. Coll. Nutr.*, **11** (1992) 340. J. L. Domingo, M. Gomez, J. M. Llobet, J. Corbella, *Kidney Int.*, **39** (1991) 598; J. L. Domingo, M. Gomez, D. J. Sanchez, J. M. Llobet, J. Corbella, *Res. Com. Chem. Pathol. Pharmacol.*, **79** (1993) 377; N. A. Partridge, F. E. Regnier, J. L. White, S. L. Hem, *Kidney Int.*, **35**, 1413-1417 (1989); P. Slanina, W. Frech, L. G. Edtrom, L. Loof, S. Slorach, A. Cedergren, *Clin. Chem.*, **32** (1986) 539.
3. W. R. Harris, *Clin. Chem.* **38** (1992) 1809; G. E. Jackson, *Polyhedron* **9** (1990) 163; P. M. May in G. Berthon (ed.), *Handbook of Metal-Ligand Interactions in Biological fluids. Bioinorganic Chemistry*, Marcel Dekker, N.Y., 1995. pp 1291-1298.
4. P. Djurdjevic, R. Jelic, *Z. Anorg. Allg. Chem.*, **575** (1989) 217.
5. E. Marklund, L. O. Ohman, *Acta Chem. Scand.*, **44** (1990) 353.
6. K. S. J. Rao, G. V. Rao, *Mol. Cell. Biochem.*, **137** (1994) 61.
7. S. B. Karweer, B. P. Pillai, R. K. Iyer, *Magnetic Res. Chem.*, **28** (1990) 922.
8. H. L. Yadava, S. Singh, P. Prasad, R. K. P. Singh, P. C. Yadava, K. L. Yadava, *Bull. Soc. Chim. Fr.*, (1984) I-314
9. S. Singh, S. Gupta, P. C. Yadava, R. K. P. Singh and K. L. Yadava, *Z. Phys. Chem. Lepzig*, **267** (1986) 902
10. T. Kiss, I. Sovago, I. Toth, A. Lakatos, R. Bertani, A. Tapparo, G. Bombi and R. B. Martin, *J. Chem. Soc. Dalton Trans.*, 1967 (1997).
11. P. Djurdjevic, R. Jelic, *Main Group Met. Chem.*, **21** (1998) 331.
12. Ph. Charlet, J. P. Deloume, G. Duc, G. Thomas-David, *Bull. Soc. Chim. Fr.*, **7-8** (1984) 222.

13. S. Dayde, Etude des équilibres de complexation et speciation simulée de la fraction ultrafiltrable de l'aluminium dans le plasma sanguin et la fluide gastro-intestinal. Implications pour la toxicité de l'aluminium. Thèse de Doctorat de l'Université Paul Sabatier, Toulouse, 1990
14. K. A. Vinnikova, C. R. Kukreja, L M. Hess, *J. Mol Cell Cardiol.*, **24** (1992) 465.; G. N. Sitton, S. J. Dixon, C. Astbury, J. R. Francis, A. H. Bird, V. Wright, *Ann Rheum Dis.*, **47** (1988) 48.; E. Ohmura-Hitomi, N. Amano, Y. Aoyama, A. Yoshida, *Lipids*, 27 (1992) 755; Y. Aoyama, T. Tsuda, E. Hitomi-Ohmura, A. Yosshida, *Int. J. Biochem.*, **24** (1992) 981; Q. Cai, G. Takemura, M. Ashraf, *J Cardiovasc. Pharmacol.*, **25** (1995) 147.
15. C. K. Mathews, K. E. van Holde, *Biochemistry*, 2nd edition, The Benjamin/Cummings Publ. Co., Menlo Park, 1995; J.A. Gelenberg, D.J. Wojcik, E.W. Falk, J.R. Baldessarini, H.S. Zeisel, D. Schonfeld, S.G. Mok, *J. Affect. Disord.* 19 (1990) 125; J.F. Rohr, D. Lobbregst, L.H. Levy, *Am. J. Clin. Nutr.* **67** (1998) 473; A. Filho-Basile, E.A. El-Khoury. L. Beaumier, Y.S. Wang, R.V. Young, *J. Am. Clin. Nutr.* **65** (1997) 473.
16. C. F. Baes and R. E. Mesmer, *The Hydrolysis of Cations*, J. Wiley & Sons, N.Y., 1976; N. B. Milic, Z. D. Bugarcic and P. Djurdjevic, *Can. J. Chem.* **69** (1991) 28; L. Ohman and W. Forsling, *Acta Chem. Scand. A.* **35** (1981) 795; P. L. Brown, R. N. Sylva, G. E. Batley and J. Ellis, *J. Chem. Soc. Dalton Trans.* 1967 (1985); J. W. Akitt, N. N. Greenwood, B. L. Khandelwahl and G. D. Lester, *J. Chem. Soc. Dalton Trans.* 604 (1972); M. Venturini and G. Berthon, *J. Chem. Soc. Dalton Trans.* 1145 (1987); M. P. Bertsch in G. Sposito (Ed.), *The Environmental Chemistry of Aluminum*, CRC, Boca Raton, 1989; R. B. Martin, *J. Inorg. Biochem.*, **44** (1991) 141; R. B. Martin, *Acc. Chem. Res.*, **27** (1994) 204.
17. P. W. Linder, R. G. Torington and D. R. Williams, *Analysis Using Glass Electrode*, Open University Press, Milton Keynes, England, 1984.
18. P. Gans, A. Sabatini and A. Vacca, *J. Chem. Soc. Dalton Trans.* 1195 (1985)
19. E. Martell and R. J. Motekaitis, *Determination and Use of Stability Constants*, VCH, Weinheim, 1988.
20. E. Martell and R. M. Smith, *Critical Stability Constants, Vol. 1, Amino Acids*, Plenum Press, New York, 1974; A. E. Martell and R. M. Smith, *Critical Stability Constants*, Vol 5, First Supplement, Plenum Press, New York, 1982.
21. R. J. Abraham, J. Fisher, P. Loftus, *Introduction to NMR Spectroscopy*, J. Wiley & Sons, 1988. pp. 232 – 235; C. C. McDonald, W. D. Phillips, *J. Am Chem. Soc.*, **85** (1963) 3736; H. Aiba, Y. Kuroda, H. Tanaka, *Bull. Chem. Soc. Jpn.*, **49** (1976) 1313; B. Noszal, D. Rabenstein, *J. Phys. Chem.*, 95 (1991) 4761; R. Bruce Martin, R. Mathur, *J. Am. Chem. Soc.*, **87** (1965) 1065; J. W. Akitt, *Prog. Nucl. Mag. Res. Spectr.* **21** (1989) 1
22. Hyperchem. Release 6.01. Professional version for Windows, Molecular Modeling System, Hypercube Inc., Canada, 1999-2000.
23. D. J. Leggett (ed), *Computational Methods for the Determination of Formation Constants*, Plenum Press, New York, 1985.

**Received: November 8, 2001 – Accepted: November 21, 2001 – Accepted in publishable format: January 23, 2002**

